# Oxysophocarpine Prevents the Glutamate-Induced Apoptosis of HT–22 Cells via the Nrf2/HO–1 Signaling Pathway

**DOI:** 10.3390/cimb46110777

**Published:** 2024-11-16

**Authors:** Ruiying Yuan, Dan Gao, Guibing Yang, Dongzhi Zhuoma, Zhen Pu, Yangzhen Ciren, Bin Li, Jianqing Yu

**Affiliations:** 1 Department of Pharmaceutical Sciences, School of Medicine, Tibet University, Lhasa 850000, China; 1yuan2yuan@163.com (R.Y.); gaodan2677591994@163.com (D.G.); yangguibin0910@163.com (G.Y.); 17844614104@163.com (D.Z.); 18237623635@126.com (Z.P.); lichangyu19971204@163.com (Y.C.); 2 College of Pharmacy, Wuhan University, Wuhan 430000, China; jq_yu@126.com

**Keywords:** oxysophocarpine, oxidative stress, Nrf2/HO–1 signal pathway, apoptosis

## Abstract

Oxysophocarpine (OSC), a quinolizidine alkaloid, shows neuroprotective potential, though its mechanisms are unclear. The aim of the present study was to investigate the neuroprotective effects of OSC through the nuclear factor erythroid 2−related factor 2 (Nrf2)/ heme oxygenase−1 (HO–1) signaling pathway using the HT–22 cell line. Assessments of cell viability were conducted utilizing the 3−(4,5−dimethylthiazol−2−yl)−2,5−diphenyltetrazolium bromide (MTT) assay. Assessments of oxidative stress (OS) were conducted through the quantification of reactive oxygen species (ROS). The integrity of the mitochondrial membrane potential (MMP) was scrutinized using fluorescent probe technology. Apoptosis levels were quantified using terminal deoxynucleotidyl transferase dUTP nick end labeling (TUNEL) staining. The trafficking of Nrf2 within the cell nucleus was examined through immunofluorescence analysis. Furthermore, Western blotting (WB) was applied to evaluate the expression levels of proteins implicated in apoptosis and the Nrf2/HO–1 pathway. To further probe the influence of OSC on the overexpression of antioxidant enzymes, cells were subjected to transfection with HO–1 siRNA. The results showed that OSC inhibited glutamate-induced OS, as evidenced by reduced cell viability and ROS levels. Furthermore, the apoptotic condition induced by glutamate in HT–22 cells was significantly reduced following OSC treatment. More interestingly, the Nrf2/HO–1 signaling pathway was upregulated following OSC treatment. These results suggest that OSC can exert neuroprotective effects by regulating the Nrf2/HO–1 pathway to inhibit neuronal cell apoptosis, potentially aiding in the treatment of neurodegenerative diseases.

## 1. Introduction

The HT–22 cell line, derived from the mouse hippocampus, serves as a valuable model for studying the intricate dynamics of neuronal function and dysfunction. This cell line is a preferred model for investigating oxidative glutamate toxicity that operates independently of receptor mediation [1,2]. Glutamate, being the quintessential neurotransmitter that elicits a stimulating response within the central nervous system, is instrumental in facilitating standard neural communication, fostering development, guiding neuronal differentiation, and enhancing synaptic plasticity [3,4]. However, an overabundance of extracellular glutamate can precipitate neuronal depolarization, a detrimental phenomenon termed excitotoxicity, which can culminate in neuronal demise. Neurodegenerative diseases are often associated with excitotoxicity (such as Alzheimer’s disease) [5,6,7,8]. The HT–22 cell line, though devoid of the ionotropic glutamate receptor (iCluR), displays sensitivity to heightened levels of extracellular glutamate. An excess of glutamate can invert the function of the Cystine (CySS)/glutamate antiporter, known as system Xc-, hindering the uptake of CySS. This functional reversal can escalate the depletion of CySS within neurons, eventually draining GSH (glutathione) reserves and causing an accumulation of free radicals. This sequence elucidates how, devoid of glutamate receptors, the cells can still succumb to glutamate-induced toxicity. This toxicity is precipitated by a Ca^2+^−independent, non−receptor−mediated oxidative mechanism associated with the reversal of the glutamate transporter’s function [9,10,11].

Oxidative stress (OS) represents a physiological condition where there is an imbalance between the generation of reactive oxygen species (ROS) within cellular compartments and the capacity to counteract these reactive entities or to restore cellular integrity following damage [12,13,14,15]. ROS, which are byproducts inevitably produced during the metabolic processes of normal cells, molecules such as superoxide anions and hydroxyl radicals, which are pivotal in cellular signaling and the maintenance of cellular balance, can also contribute to OS under certain conditions. However, excessive ROS production or impaired antioxidant defense mechanisms can lead to OS. OS-induced cellular changes encompass a wide range of molecular alterations, affecting membrane stability, protein function, Deoxyribonucleic Acid (DNA) integrity, and cellular signaling [16,17,18,19]. These alterations can have significant implications for cellular homeostasis and may ultimately contribute to the development of a range of diseases. Therefore, understanding and modulating OS is a critical area of research in the pursuit of therapeutic strategies for numerous health conditions.

The nuclear factor erythroid 2−related factor 2 (Nrf2)/ heme oxygenase−1 (HO–1) signaling pathway serves a protective function in cells under OS and when the redox homeostasis is disrupted, serving as a critical cellular defense mechanism [20]. Nrf2 can regulate antioxidant response elements (AREs) within the genome and acts as a transcription factor within the cell [21,22]. Under specific conditions, such as exposure to OS or electrophilic compounds, Nrf2 can be activated and transferred to the nucleus from the cytoplasm. Upon entering the nucleus, Nrf2 binds to AREs and triggers the transcription of a series of protective genes, including HO–1 [23,24,25]. Upon activation, HO–1 binds to specific DNA sequences, thereby initiating the transcription of the HO–1 gene. Subsequently, HO–1 is translated into a protein and trafficked to the cytosol, where it acts as an enzyme to degrade heme into biliverdin, carbon monoxide, and iron, providing defense against oxidative damage, inflammatory injury, and apoptosis within the cell [26,27]. The Nrf2/HO–1 signaling pathway may be a critically important target in the search for drugs to treat neurodegenerative diseases caused by oxidative damage and cell apoptosis. Additionally, the dysregulation of this pathway is associated with the pathogenesis of various diseases, making its modulation highly promising for potential therapeutic intervention.

Oxysophocarpine (OSC) is an oxidation product of sophocarpine, belonging to the saponin alkaloid class and meticulously extracted from the roots of Sophora species, which has garnered significant attention in natural product research [28]. Previous research has shown that OSC exhibits a range of biological activities, such as anti−inflammatory, antioxidant, neuroprotective, and anticancer properties [29,30,31,32]. In addition to in vitro studies, several in vivo studies have also investigated the antioxidant potential of OSC. For instance, a study by Liu C et al. demonstrated that OSC administration significantly reduced oxidative stress markers and improved antioxidant enzyme activity [33]. Another study by Shi X et al. showed that OSC enhanced the antioxidant capacity of the tissues surrounding the osteolytic area [34]. These in vivo findings support the notion that OSC may have a protective role against oxidative stress-related pathologies. Due to its unique chemical structure and potent biological activities, OSC exhibits strong potential as a candidate drug for the treatment of various diseases. In particular, recent studies have concentrated on the neuroprotective properties of OSC, highlighting its potential in the management of neurodegenerative diseases characterized by OS and apoptosis [28]. Despite the known multifaceted biological activities of OSC, its specific contribution to neuroprotection remains incompletely understood. The aim of the present study was to investigate the neuroprotective effects of OSC in HT–22 cells and to elucidate the underlying mechanisms, thereby enhancing our understanding of its potential role in the drug discovery and development process for neurodegenerative disorders.

## 2. Materials and Methods

### 2.1. Materials and Reagents

Oxysophocarpine (purity > 98%) was sourced from Sichuan Weikeqi Biotechnology Co., Ltd., Chengdu, China, with the CAS number 26904−64−3. All reagents were purchased from Sigma−Aldrich (St. Louis, MO, USA). Cell culture essentials, such as Dulbecco’s Modified Eagle’s Medium (DMEM) fortified with 10% fetal bovine serum (FBS) and phosphate-buffered saline (PBS), were sourced from GIBCO (Grand Island, NY, USA). A range of primary and secondary antibodies, including those against HO–1, Nrf2, Bcl−2−associated X protein (BAX), B−cell lymphoma 2 (BCL–2), cysteine aspartate protease 3 (caspase–3), cysteine aspartate protease 9 (caspase–9), cleaved caspase–3, and cleaved caspase–9, were sourced from Affinity Biosciences (Watertown, MA, USA). The inhibitor of HO–1 activity, tin protoporphyrin IX (SnPP), and its inducer, cobalt protoporphyrin IX (CoPP), along with siRNA against HO–1, were obtained from Abcam (Cambridge, UK). The 2′,7′−dichlorofluorescin diacetate (DCFH−DA), mitochondrial membrane potential (MMP) assay kit (JC−1), and the terminal deoxynucleotidyl transferase dUTP nick end labeling (TUNEL) apoptosis assay kit were all procured from Beyotime Biotechnology (Shanghai, China). The Bradford protein assay kit was purchased from Solarbio Science & Technology (Beijing, China).

### 2.2. Cell Culture

HT–22 cells were obtained from the China Center for Type Culture Collection (Wuhan, China) and cultured in DMEM supplemented with 10% FBS and 1% penicillin−streptomycin (P/S; Millipore, Burlington, MA, USA). HT–22 cells were nonmature neuroblasts. The cells were cultured in a humidified atmosphere of 37 °C and 5% CO_2_ for 24 h and then used at 80% confluence.

### 2.3. Assay of the Appropriate Concentrations of OSC

The impact of various concentrations of OSC on cell viability was assessed using the 3−(4,5−dimethylthiazol−2−yl)−2,5−diphenyltetrazolium bromide (MTT) assay. HT–22 cells were incubated for 24 h and then seeded at a density of 8 × 10^3^ cells per well in 96−well culture plates. The cells were then exposed to concentrations of OSC ranging from 1.25 to 20 μM (at double intervals) for 12 h. Following this, MTT assays were performed to evaluate the effect of OSC on cell viability. Detailed procedures for the MTT assay are provided in Section 2.4.

### 2.4. Cell Viability Assay

The alterations in cell viability were evaluated using the MTT assay. HT–22 cells were cultured for 24 h and then seeded at a density of 8 × 10^3^ cells per well in 96-well culture plates. The cells were subsequently exposed to a range of OSC concentrations from 1.25 to 10 μM (at double intervals) for 12 h. The positive drug was treated with 50 μM trolox as a control group and then cultured with 20 mM glutamate for 24 h to induce the model. Subsequently, 50 μL of MTT solution at a concentration of 2.5 mg/mL was added to each well and the culture continued for an additional 4 h. Finally, 150 μL of dimethyl sulfoxide (DMSO) was added to dissolve the formazan crystals, and the optical density (OD) at 490 nm was measured using an enzyme-linked immunosorbent assay (ELISA) plate reader (Infinite M Plex, Shanghai, China). The cell viability rates were calculated.

### 2.5. Assay of Intracellular ROS Production

The level of ROS generation was determined using DCFH−DA. HT–22 cells were incubated for 24 h and then seeded at a density of 1 × 10^5^ cells per well in 24−well culture plates, followed by administration as described in Section 2.4. Subsequently, the cells were incubated with a diluted DCFH−DA (10 μM/L) probe for 20 min, followed by three washes with PBS, and the fluorescence intensity of DCF was measured at 495/517 nm (excitation/emission) using an ELISA plate reader and calculated.

### 2.6. MMP Determination

The mitochondria were stained with the fluorescent probe JC-1 to assess the MMP. Cells of the HT–22 cell line were cultured for 24 h before being seeded into a 6−well plate at a density of 3.1 × 10^5^ cells per well. The cells were subsequently treated with concentrations of OSC ranging from 1.25 to 10 μM (at double intervals) for 12 h, then cultured with 20 mM glutamate for 24 h to induce the model. Subsequently, the cells were rinsed with PBS and cultured with diluted JC−1 dye for 30 min. The supernatant was then mixed with the medium, and a fluorescence microscope was employed to visualize the cells. Mitochondrial depolarization could be marked using green fluorescence, whereas mitochondrial polarization was indicated by red fluorescence. The ratio of the intensities of these two types of fluorescence was utilized to assess the degree of mitochondrial depolarization. The quantities of red and green fluorescence were quantified and calculated relative to each other using the ImageJ 1.54f software, from which the rate of mitochondrial depolarization was derived.

### 2.7. Apoptosis Detection

The single−use TUNEL assay kit was employed to quantify apoptotic cell death. HT–22 cells were subjected to the treatment protocol outlined in Section 2.6. Subsequently, the cells were fixed in a 4% paraformaldehyde solution for 30 min, after which they were washed with PBS. The cells were then subjected to permeabilization using 0.3% Triton X−100 at ambient temperature for 5 min, followed by another rinse with PBS. Following this, the cells were incubated with the TUNEL detection reagent at 37 °C for 60 min. After washing with PBS, the cells were mounted using a fluorescence−blocking mounting medium containing 4′,6−diamidino−2−phenylindole (DAPI) for nuclear staining. The samples were examined under a fluorescence microscope. Apoptotic cells exhibited green fluorescence due to TUNEL staining, while the nuclei displayed blue fluorescence from DAPI staining. The proportion of TUNEL−positive cells was determined, and the percentage of apoptotic cells was calculated based on this ratio. Randomly selected TUNEL−positive cells from ten distinct fields of view, each at the same magnification, were analyzed. ImageJ software was utilized to assess the fluorescence intensity, facilitating the quantification of apoptosis rates.

### 2.8. Western Blot Analysis

The protein expression levels were meticulously assessed through a series of standardized biochemical procedures. Following the guidelines provided by the manufacturer (Pierce Biotechnology, Rockford, IL, USA), the extraction of both nuclear and cytoplasmic proteins was performed. The total protein content was subsequently quantified using the Bradford protein assay kit, ensuring accurate quantification. The cell lysate, with equivalent amounts of protein, was subjected to sodium dodecyl sulfate−polyacrylamide gel electrophoresis (SDS−PAGE). Gels with a gradient concentration of 10% to 15% were employed to resolve proteins according to their molecular weight, after which the resolved proteins were transferred onto a nitrocellulose membrane. The membrane underwent blocking with 1× TBST buffer supplemented with 5% skimmed milk powder at 4 °C overnight and was washed three times with 1× TBST buffer. The membrane was subsequently exposed to a primary antibody panel, including those directed against BAX, Bcl−2, Nrf2, HO–1, caspase–3, and other relevant markers, each diluted at a ratio of 1:1000. This antibody incubation was conducted in a blocking solution at room temperature for 1.5 h, followed by washing to eliminate unbound antibodies. The membrane was then incubated with either goat anti−rabbit or rabbit anti−mouse antibodies, each diluted at a ratio of 1:5000, and incubated with horseradish peroxidase for 1 h. Detailed antibody information is shown in Table 1. The intensity of immunoreactive bands was quantified using a ChemiDoc image analyzer (Tanon 4600, Tanon, Shanghai, China) after they were visualized with ECL Western Blotting (WB) Substrate (Amersham Bioscience, Buckinghamshire, UK).

### 2.9. Fluorescence Staining Analysis

The subcellular localization of Nrf2 was examined through an immunofluorescence approach to discern its nuclear translocation. HT–22 cells were cultivated on Lab−Tek II chamber slides, providing an optimal environment for cellular adherence and growth. Following treatment with OSC, cells were fixed using formaldehyde to preserve their structural integrity and then permeabilized with cold acetone to facilitate antibody penetration. The cells were detected with a secondary antibody that identified Nrf2 (Santa Cruz Biotechnology, Dallas, TX, USA), which was fluorescently labeled with fluorescein isothiocyanate (FITC) (Alexa Fluor 488; Invitrogen, Carlsbad, CA, USA). The nuclei were observed by applying PBS with DAPI at a concentration of 1 mg/mL and 50 μL of VectaShield to the cells (Vector Laboratories, Burlingame, CA, USA). A Provis AX70 fluorescence microscope (Olympus Optical, Tokyo, Japan) was used to observe and capture the stained cells.

### 2.10. Transfection of siRNA Targeting HO–1

The transfection protocol for small interfering RNA (siRNA) specific to HO–1, provided by Invitrogen (Carlsbad, CA, USA), was followed. The primer sequences were as follows: HO–1, sense (5′-3′) CUGCUCAACAUUGAGCUGUTT and antisense (5′-3′) ACAGCUCAAUGUUGAGCAGTT. HO–1 siRNA was introduced into HT–22 cells for 48 h before stimulation with OSC according to the manufacturer’s guidelines. Cells were seeded in 24−well plates at a density of 4 × 10^4^ cells per well, allowing them to reach a confluence of 30–50% for optimal transfection efficiency. Cells were transfected with 33 nM of a negative control siRNA or an siRNA targeting HO–1, which was incorporated into a plasmid, using Opti−DMEM supplemented with lipofectamine and serum (Gibco, Grandisland, NY, USA). Following transfection, the cells were incubated in DMEM for recovery. The expression levels of target proteins in the transfected cells after 48 h were subsequently assessed. Cells that had successfully undergone transfection were selected for further experiments assessing cell viability and ROS levels.

### 2.11. Statistical Analysis

The data are presented as the mean ± standard deviation (SD). Each experiment was conducted at least three times to ensure reproducibility. Statistical analysis was performed using GraphPad Prism version 6.0 (GraphPad Prism Software Inc., San Diego, CA, USA). The data were subjected to one−way analysis of variance (ANOVA) followed by Tukey’s post hoc test to identify statistically significant differences (*p* < 0.05).

## 3. Results

### 3.1. Determining the Appropriate Concentrations of OSC

**Figure 1:**
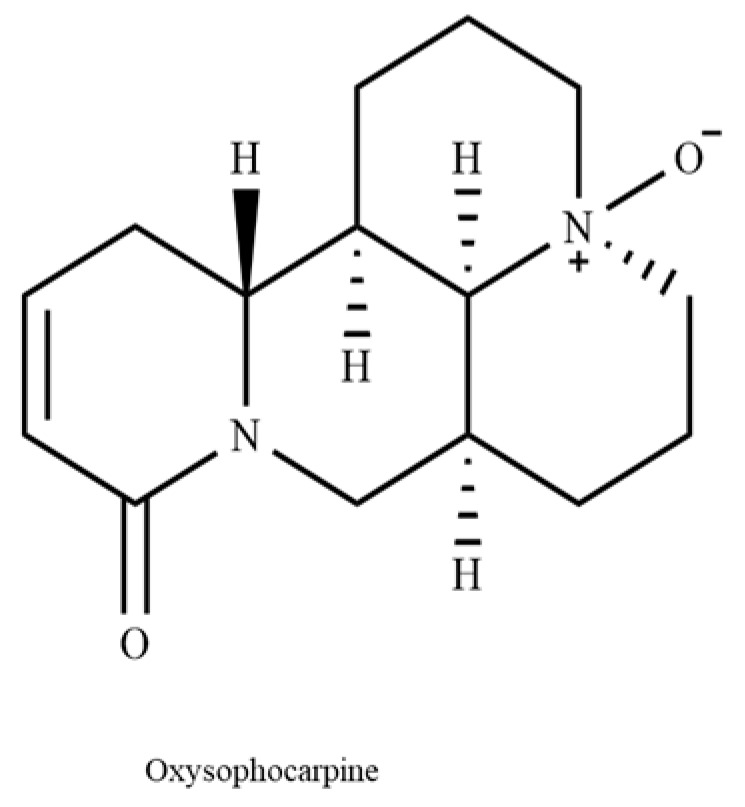
Molecular structure of oxysophocarpine.

**Figure 2:**
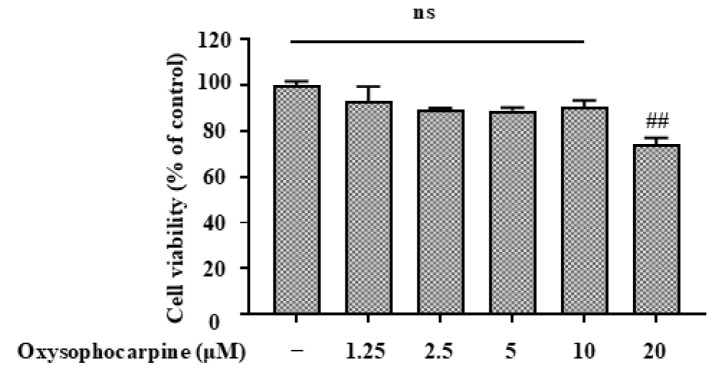
The effects of OSC on cell viability in HT–22 cells were assessed. HT–22 cells were exposed to varying concentrations (1.25, 2.5, 5, 10, 20 μM) of OSC for a period of 12 h. Cell viability was determined using the MTT assay. Each bar in the graph represents the mean ± standard deviation (SD), derived from three independent experiments (*n* = 3). “ns” stands for “not significant”, The bars marked with ## indicate a statistically significant difference compared to the control group (*p* < 0.01).

To determine the appropriate concentration of OSC (Figure 1) and eliminate the negative effects of subsequent research on OSC, the viability of cells incubated with OSC was determined using MTT. A total of 20 μM of OSC produced cytotoxicity to HT–22 cells, and cell viability decreased to 74.02% ± 3.17%. However, 1.25, 2.5, 5, and 10 μM of OSC did not affect the cell viability of HT–22, and these concentrations were selected for subsequent experimental studies (Figure 2).

### 3.2. The Influence of OSC on Glutamate-Induced Cytotoxicity and ROS Production in HT–22 Cells Was Assessed

**Figure 3:**
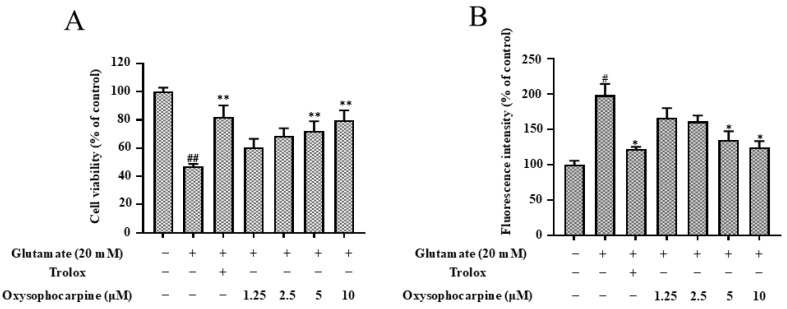
The influence of OSC on glutamate-induced cytotoxicity and ROS production in HT–22 cells was assessed. Prior to a 24 h exposure to glutamate at a concentration of 20 mM, HT–22 cells were subjected to pretreatment with a range of OSC concentrations (1.25, 2.5, 5, 10 μM). Panel (**A**) illustrates the assessment of cell viability utilizing the MTT assay, while Panel (**B**) depicts the quantification of ROS production using the DCF Fluorescence intensity. Trolox, administered at 50 μM, served as a benchmark for a positive control. The data are presented as a percentage relative to untreated cell populations, with each bar signifying the mean ± SD derived from triplicate experiments. Statistical significance is denoted as follows: ^#^ *p* < 0.05 and ^##^ *p* < 0.01 in contrast to the untreated control group; * *p* < 0.05 and ** *p* < 0.01 in contrast to the group exposed solely to 20 mM glutamate. The presence or absence of treatments is indicated by “+” and “−”, respectively. ROS, reactive oxygen species.

After glutamate induction, HT–22 cells produced OS, which reduced cell viability and increased ROS production. The cell viability of HT–22 cells exposed to 20 μM glutamate decreased to 46.98% ± 1.85%; after trolox treatment, cell viability recovered to 81.92% ± 8.38%. Following exposure to varying concentrations of OSC, cell viability increased and was concentration−dependent, with the strongest protective effect at 10 μM, where cell viability recovered to 79.67% ± 7.17% (*p* < 0.01). Therefore, OSC can inhibit the toxicity of glutamate-induced HT–22 cells (Figure 3A). After treatment with 20 μM glutamate, the average oxidation DCF peak intensity of the model group cells increased by about 1.98 times compared with that of the untreated control group cells; after trolox treatment, the average oxidation DCF peak intensity of the model group cells treated with glutamate decreased by 0.39 times; pretreatment with OSC reduced ROS accumulation induced by glutamate in a dose−dependent manner, with the strongest inhibitory effect at 10 μM (*p* < 0.05) (Figure 3B).

### 3.3. Effects of OSC on Glutamate-Induced Mitochondrial Function in HT–22 Cells

**Figure 4:**
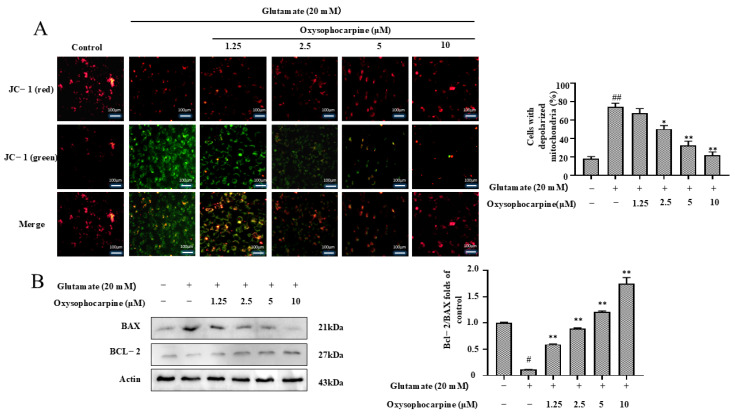
The impact of OSC on the modulation of the MMP and the expression of the apoptotic proteins BCL–2/BAX in glutamate-exposed HT–22 cells was investigated. HT–22 cells were pretreated with a range of concentrations (1.25, 2.5, 5, 10 μM) of OSC, followed by a 24 h exposure to glutamate at a concentration of 20 mM. (**A**) The MMP was evaluated using JC−1 staining, which was observed under a microscope at 200× magnification. Green fluorescence indicated mitochondrial depolarization, whereas red fluorescence represented normal polarization. (**B**) The levels of BCL–2/BAX were quantified through Western blotting (WB), with the expression levels normalized against actin as a loading control. The data, represented as mean values ± SD, were derived from three independent experiments (n = 3). Statistical significance is denoted as follows: ^#^ *p* < 0.05 and ^##^ *p* < 0.01 indicate significant differences compared to the untreated control; * *p* < 0.05 and ** *p* < 0.01 represent substantial differences from the group treated with glutamate alone (20 mM). The symbols “+” and “−” represent the inclusion or exclusion of the respective treatments.

To evaluate the impact of OSC on mitochondrial function in HT–22 cells subjected to glutamate induction, we measured the MMP and the levels of BCL–2/BAX expression. After treatment with 20 μM glutamate, the number of depolarized mitochondria increased, but pretreatment with different concentrations of OSC significantly reduced the number of depolarized mitochondria (Figure 4A). Moreover, a decrease in the ratio of Bcl−2 to BAX expression levels can alter the permeability of the mitochondrial membrane, thereby increasing the levels of apoptotic activators. Following treatment with 20 μM glutamate, the ratio of Bcl−2 to BAX expression levels decreased in HT–22 cells. Compared to HT–22 cells treated with glutamate, pretreatment with different concentrations of OSC increased the ratio of Bcl−2 to BAX expression levels, and this effect was concentration−dependent, with a significant increase in the ratio of Bcl−2 to BAX expression levels observed at 10 μM OSC (*p* < 0.05) (Figure 4B).

### 3.4. Impact of OSC on Apoptosis in HT–22 Cells Induced by Glutamate

**Figure 5:**
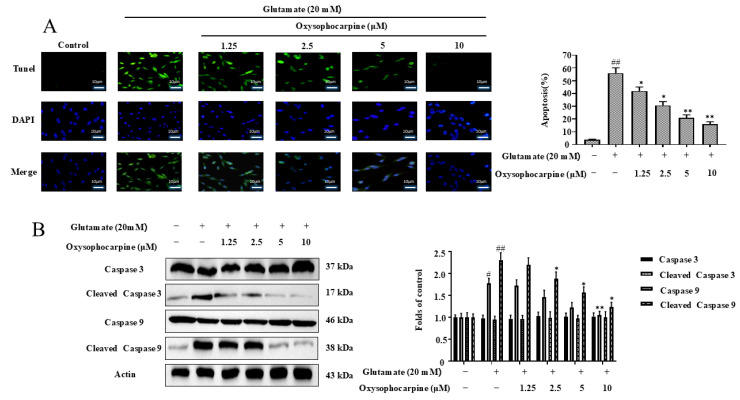
This study investigates the impact of OSC on the apoptotic response in HT-22 cells following glutamate exposure. (**A**) The apoptotic rate in HT–22 cells, subjected to 20 mM glutamate for 24 h with a prior treatment of OSC at concentrations of 1.25, 2.5, 5, and 10 μM, was ascertained using the TUNEL staining method. Apoptotic cells were identified by green fluorescence under a 200× microscope magnification. (**B**) The levels of apoptosis-related proteins, including cleaved caspase–3, caspase–3, cleaved caspase–9, and caspase–9, were assessed via WB analysis. The expression data were normalized against actin, a constitutively expressed protein. The results are expressed as a percentage relative to the control cells, which were not treated. Each bar graph displays the mean ± SD from three independent experiments (n = 3). Statistical significance is indicated as follows: ^#^ *p* < 0.05 and ^##^ *p* < 0.01 indicate significant differences from the untreated control; * *p* < 0.05 and ** *p* < 0.01 suggest substantial differences from the group treated solely with 20 mM glutamate. The inclusion or exclusion of treatments is indicated by “+” and “−”, respectively.

The purpose of this study was to ascertain the potential of OSC in mitigating apoptosis in HT–22 cells triggered by glutamate. A comprehensive analysis was conducted to evaluate the apoptotic rate and the expression levels of critical apoptotic proteins, including cleaved caspase–3, caspase–3, cleaved caspase–9, and caspase–9. In our experiments, HT–22 cells exposed solely to glutamate exhibited a marked increase in green fluorescence intensity, indicative of heightened apoptosis, reaching a rate of 55.99% ± 4.11% (*p* < 0.01). This finding underscores the pro−apoptotic effect of glutamate. Conversely, the pretreatment of HT–22 cells with OSC at varying concentrations led to a notable reduction in fluorescence intensity and a subsequent decrease in the apoptotic rate. Notably, at a concentration of 10 µM OSC, the apoptotic rate was significantly lowered to 15.88% ± 2.01% (*p* < 0.01), demonstrating a concentration−dependent protective effect of OSC against glutamate−induced apoptosis. Furthermore, when HT–22 cells were treated with 20 μM glutamate in isolation, no significant alterations were observed in the expression levels of caspase–3 and caspase–9. In contrast, the levels of their activated forms, cleaved caspase–3 and cleaved caspase–9, were substantially elevated (*p* < 0.05), highlighting the activation of apoptotic pathways. Co−treatment with OSC, however, attenuated this effect, with a gradual and concentration−dependent decrease in the expression levels of the cleaved forms. At the optimal concentration of 10 μM OSC, the expression of cleaved caspase–3 and cleaved caspase–9 was significantly diminished (*p* < 0.05), further substantiating the neuroprotective role of OSC. This illustrates the capacity of OSC to modulate the apoptotic response in HT–22 cells, potentially through the regulation of caspase activation, thereby offering a promising therapeutic avenue for neurodegenerative conditions associated with excitotoxicity (Figure 5A,B).

### 3.5. Effects of OSC on Nrf2 Translocation in HT–22 Cells

**Figure 6:**
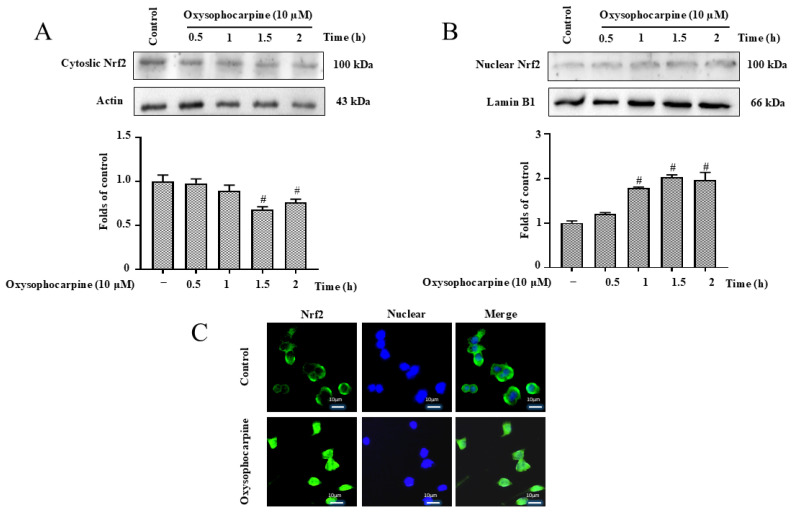
Influence of OSC on Nrf2 translocation dynamics in HT–22 cells. The study examines the effect of OSC on the subcellular distribution of Nrf2 in HT–22 cells following exposure to a concentration of 10 μM for intervals of 0.5, 1, 1.5, or 2 h. (**A**,**B**) Nrf2 protein levels in both cytosolic and nuclear compartments were ascertained by WB analysis. This approach allows for the assessment of Nrf2 translocation from the cytoplasm to the nucleus in response to OSC treatment. (**C**) The visualization and quantification of Nrf2 translocation were further accomplished using immunofluorescence microscopy, providing a qualitative representation of protein movement within the cellular context. For the normalization of protein levels, cytosolic Nrf2 was referenced against actin, while nuclear Nrf2 was calibrated against lamin B1, ensuring the accuracy of the comparative analysis. Data are depicted as the mean ± SD derived from three independent experiments (n = 3). Statistical significance is represented by the following notations: ^#^ *p* < 0.05 indicate significant differences when compared to the control group without treatment.

Nrf2 Translocation as a Regulator of OS Inhibition by OSC in HT–22 Cells. Nrf2 translocation is recognized as a pivotal pathway in the cellular defense against OS. This study aimed to scrutinize the influence of OSC on the translocation dynamics and expression levels of Nrf2 in HT–22 cells. Our approach involved quantifying the translocation and expression of Nrf2 in response to OSC treatment. To provide a comprehensive assessment, we employed both immunofluorescence and WB analysis. The immunofluorescence technique allowed us to visualize the subcellular redistribution of Nrf2, offering a qualitative perspective on the translocation event. Concurrently, WB analysis provided a quantitative measure of Nrf2 protein levels in different cellular compartments. The immunofluorescence findings were corroborated by the WB results, revealing a coherent pattern of Nrf2 translocation in response to OSC. The data collectively indicated a significant alteration in Nrf2 localization, suggesting that OSC may modulate the Nrf2 pathway, thereby potentially influencing the cell’s OS response (Figure 6A–C).

### 3.6. Effects of OSC on Expression of HO–1 in HT–22 Cells

**Figure 7:**
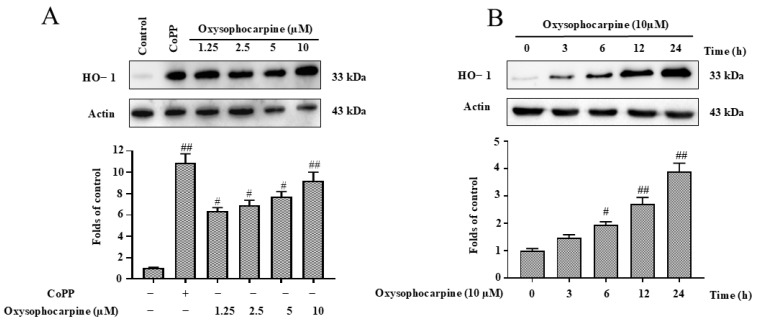
Modulation of HO–1 protein expression by OSC in HT–22 Cells. (**A**) Cells were exposed to a range of OSC concentrations (1.25, 2.5, 5, 10 μM) for a duration of 12 h to determine the dose-dependent effect on HO–1 expression. Cobalt protoporphyrin (CoPP), at a concentration of 20 μM, served as a positive control to validate the response. (**B**) To explore the time course of HO–1 induction, cells were treated with a fixed concentration of 10 μM OSC for varying periods. The protein expression of HO–1 was quantified using WB analysis, a method that allows for the detection and quantification of specific proteins. The results were normalized relative to actin, a reference protein, to adjust for any variations in protein loading. The data presentation follows the standard format, where each bar graph segment illustrates the mean ± SD from triplicate samples (n = 3), ensuring the reproducibility and reliability of the findings. Statistical significance is denoted by the symbols *^#^ p* < 0.05 and *^##^ p* < 0.01, which indicate significant differences in HO–1 expression levels when compared to the control group without treatment. The presence or absence of treatment is indicated by “+” and “−” signs, respectively.

**Figure 8:**
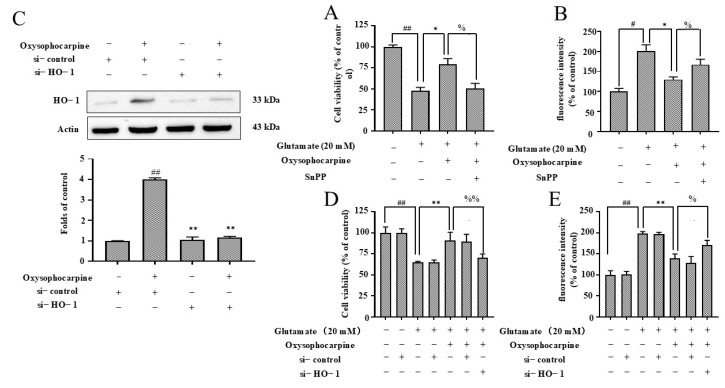
Impact of HO–1 knockdown on HT–22 cell response to OSC and glutamate challenge. This study delineates the consequences of HO–1 suppression in HT–22 cells under conditions designed to mimic OS. (**A**,**D**) The survival of HT–22 cells, following pretreatment with 10 μM OSC in conjunction with or without 50 μM SnPP and si−HO–1, was subsequently challenged with 20 mM glutamate for 24 h. The quantitative assessment of cell viability was performed using the MTT assay. (**B**,**E**) The production of ROS was evaluated through DCF fluorescence measurement, providing a quantitative assessment of intracellular ROS levels. (**C**) Representative WB images illustrate the levels of HO–1 protein expression in the treated cells, offering a visual confirmation of the HO–1 knockdown efficacy. Data are presented as the mean ± SD from three independent experiments (n = 3), ensuring the statistical robustness of the findings. Statistical significance is indicated as follows: ^#^ *p* < 0.05 and ^##^ *p* < 0.01 denote significant differences relative to the untreated control group; * *p* < 0.05 and ** *p* < 0.01 indicate substantial differences when compared to the group treated solely with 20 mM glutamate; ^%^ *p* < 0.05 and ^%%^ *p* < 0.01 signify substantial differences in comparison to the group treated with 10 μM OSC. The presence or absence of specific treatments is denoted by the symbols “+” and “−”.

Given that Nrf2 translocation can enhance HO–1 expression, CoPP was employed as a positive control for HO–1 induction, demonstrating a significant increase in HO–1 protein expression (*p* < 0.01). The treatment of HT–22 cells with OSC resulted in a concentration-dependent and time−dependent increase in HO–1 protein expression (Figure 7A,B). To evaluate the influence of OSC on the expression of HO–1 in HT–22 cells following exposure to glutamate, and its subsequent impact on cellular toxicity and ROS generation, we conducted a series of experiments to evaluate the neuroprotective potential of OSC by employing SnPP to inhibit the HO–1 pathway and by transfecting cells with the HO–1 gene. A significant elevation in cytotoxicity and ROS production was observed upon treatment with glutamate alone (*p* < 0.05). However, a substantial diminution in these effects was observed when glutamate was concurrently administered with OSC, with the difference being statistically significant (*p* < 0.05) (Figure 8A,B). Consequently, the administration of SnPP significantly reversed the inhibitory impact of OSC on glutamate-induced cytotoxicity and ROS production. Furthermore, we explored the protein expression levels following the transfection of the HO–1 gene, observing that HO–1 protein expression was suppressed following OSC treatment. To rule out potential side effects of transfection or RNA admixture into the cells, HT–22 cells were treated with siRNA against HO–1 (si−HO–1) or empty siRNA (si−NC) as a negative control. The cells were incubated with si−HO–1 and si−NC for 48 h. The protein expression level of HO–1 was assessed via WB in HT–22 cells post−si−HO–1 transfection, to confirm successful transfection (Figure 8C). Moreover, our findings indicated that, in the absence of si−HO–1 transfection, OSC significantly inhibited glutamate−induced cytotoxicity and ROS production (*p* < 0.05). However, this inhibitory effect was significantly diminished (*p* < 0.05) upon si−HO–1 transfection (Figure 8D,E). This suggests that the upregulation of HO–1 expression in HT–22 cells is predominantly linked to OSC. These results suggest that OSC may inhibit apoptosis and ultimately suppress apoptosis by upregulating HO–1 expression, thereby mitigating glutamate−induced cytotoxicity and ROS production in HT–22 cells.

## 4. Discussion

Apoptosis, a form of programmed cell death, is essential for maintaining tissue balance, development, and immune response [35]. Disruption in apoptotic regulation can lead to significant pathological conditions, including cancer, neurodegenerative diseases, and autoimmune disorders [36,37,38,39]. The intrinsic and extrinsic pathways are two well−characterized mechanisms through which apoptosis is initiated. Apoptosis in glutamate-induced HT–22 cells is primarily mediated by the intrinsic pathway [40,41,42,43]. The intrinsic pathway is initiated by intracellular stress signals, including DNA damage or OS, which result in impaired mitochondrial membrane permeability and the release of pro−apoptotic factors. In neurodegenerative diseases, such as Alzheimer’s and Parkinson’s disease, imbalances in apoptotic regulation contribute to neuronal loss and disease progression [44]. Modulating apoptotic pathways to promote neuronal survival and inhibit cell death has emerged as a potential therapeutic strategy for neuroprotection. Notably, the results indicate that OSC exerts a protective effect by reducing glutamate-induced cell apoptosis (Figure 5).

It is widely recognized that the prompt generation of ROS, particularly superoxide, which is elicited by glutamate, can instigate a detrimental cascade of events ultimately resulting in neuronal cell demise and the activation of apoptotic pathways [45,46]. This study has established that the exposure of HT–22 cells to glutamate triggers OS and enhances apoptotic levels. OSC inhibited glutamate-induced HT–22 cytotoxicity (Figure 3A). ROS is a redox messenger of intracellular signals, and the overproduction of ROS can trigger OS, the loss of cellular function, and even apoptosis [47,48,49,50]. In this study, ROS levels could be significantly increased after glutamate action on HT–22 cells, whereas OSC pretreatment reduced ROS production (Figure 3B). Therefore, OSC may protect HT–22 cells from the toxic effects of glutamate-induced cell death by inhibiting ROS production. Notably, our study reveals that OSC, an antioxidative compound, effectively ameliorates the OS provoked by glutamate, presenting a promising result.

Furthermore, the accumulation of damaged neuronal mitochondria is a characteristic feature of neurodegenerative diseases. The MMP is commonly used to measure mitochondrial function. A reduced MMP indicates mitochondrial depolarization, which can lead to mitochondrial dysfunction [51,52,53]. Bcl−2 and BAX regulate apoptotic activators by modulating the permeability of the mitochondrial membrane, which can trigger apoptosis. A reduction in the ratio of Bcl−2 to BAX expression levels promotes apoptosis, whereas an increase can inhibit apoptosis [54,55,56,57,58]. In this study, pretreatment with OSC inhibited the mitochondrial membrane disorder caused by mitochondrial depolarization with a decrease in the ratio of Bcl−2 to BAX expression levels after glutamate action in HT–22 cells (Figure 4A,B). This decrease in the ratio of Bcl−2 to BAX expression levels enhanced the release of apoptotic activators. Whereas caspase–9 is involved in the initiation of apoptosis, caspase–3 plays the role of apoptosis executor. Caspase−3 and caspase–9 cleavage activation is followed by cleaved caspase–3 and cleaved caspase–9, which in turn leads to apoptosis [59,60,61,62]. Surprisingly, the results of the study showed that OSC pretreatment reduced apoptosis by slowing down mitochondrial dysfunction and decreasing the activation of caspase–3 and caspase–9, while decreasing apoptosis after glutamate action in HT–22 cells (Figure 5B). Consequently, OSC might safeguard HT–22 cells against glutamate-mediated apoptosis by lessening mitochondrial membrane potential loss, boosting the ratio of Bcl−2 to BAX expression levels, and reducing the activity of caspase–3 and caspase–9.

The Nrf2/HO–1 signaling pathway is recognized as a pivotal element in the antioxidative defense mechanism, as it regulates the transcription of genes that encode antioxidant and detoxifying enzymes in response to OS [63,64]. The activation of Nrf2 and the induction of HO–1 expression have been associated with enhanced cellular resistance to OS and improved outcomes in various disease models. Targeting this pathway through pharmacological agents or natural compounds holds promise for the development of novel antioxidative therapies [65,66]. Natural products are favored by researchers for their mild action; in recent years, natural products have been involved in exploring more effective drug treatments for managing neurodegenerative diseases. It has been previously reported in the literature that OSC exhibits a protective function against apoptosis in airway epithelial cells. However, the mechanism of inhibition of apoptosis in neuronal cells was not clear. The results of the study showed that OSC pretreatment activated the Nrf2/HO–1 pathway, promoted the nuclear translocation of Nrf2, upregulated the protein expression level of HO–1, which inhibited ROS production, and then exerted an antioxidant effect, and ultimately inhibited the glutamate-induced apoptosis of HT–22 cells (Figure 6, Figure 7 and Figure 8). So, this study showed that OSC inhibited apoptosis in HT–22 cells induced by glutamate via activating the Nrf2/HO–1 pathway and alleviating OS and mitochondrial membrane disorders, thus exerting its neuroprotective effects.

## 5. Conclusions

This study investigated the effects of OSC on neuronal apoptosis and its mechanisms. The data elucidated that OSC can inhibit apoptosis in HT–22 cells induced by glutamate by activating the Nrf2/HO–1 pathway to alleviate OS and mitochondrial membrane disorders, thus exerting its neuroprotective effect. However, this study only demonstrated that OSC inhibited apoptosis in HT–22 cells induced by glutamate in vitro, but it remains to be investigated whether it also inhibits apoptosis in vivo. While the study explored the Nrf2/HO–1 signaling pathway, other potential mechanisms of OSC’s neuroprotective effects were not investigated. We will also continue to enrich this study in the future.

## Figures and Tables

**Table 1:** Antibody information.

Antibodies	Hosts	MW (kDa)	Dilutions	Cat.	Sources
β−actin antibody	Rabbit polyclonal antibody	43	1:1000	#AF7018	Affinity Biosciences
Lamin B1 antibody	Rabbit polyclonal antibody	66	1:1000	#AF5161	Affinity Biosciences
BAX antibody	Rabbit polyclonal antibody	21	1:1000	#AF0120	Affinity Biosciences
BCL2 antibody	Rabbit polyclonal antibody	27	1:1000	#AF6139	Affinity Biosciences
Caspase−3 antibody	Rabbit polyclonal antibody	37	1:1000	#DF6879	Affinity Biosciences
Cleaved caspase–3 antibody	Rabbit polyclonal antibody	17	1:1000	#AF7022	Affinity Biosciences
Caspase−9 antibody	Rabbit polyclonal antibody	46	1:1000	#AF6348	Affinity Biosciences
Cleaved caspase–9 antibody	Rabbit polyclonal antibody	38	1:1000	#AF5244	Affinity Biosciences
Nrf2 antibody	Rabbit polyclonal antibody	100	1:1000	#AF0639	Affinity Biosciences
HO–1 antibody	Rabbit polyclonal antibody	33	1:1000	#AF5393	Affinity Biosciences
Goat anti−rabbit IgG (H + L) HRP	_	_	1:5000	#S0001	Affinity Biosciences

## Data Availability

The original contributions presented in this study are included in the article. Further inquiries can be directed to the corresponding author.

## References

[1] Prasansuklab A., Sukjamnong S., Theerasri A., Hu V.W., Sarachana T., Tencomnao T. (2023). Transcriptomic Analysis of Glutamate-Induced HT22 Neurotoxicity as a Model for Screening Anti-Alzheimer’s Drugs. Sci. Rep..

[2] Zhang Y., Chen S., Fan F., Xu N., Meng X.-L., Zhang Y., Lin J.-M. (2023). Neurotoxicity Mechanism of Aconitine in HT22 Cells Studied by Microfluidic Chip-Mass Spectrometry. J. Pharm. Anal..

[3] Wang X., Zhang W., Ge P., Yu M., Meng H. (2022). Parthanatos Participates in Glutamate-Mediated HT22 Cell Injury and Hippocampal Neuronal Death in Kainic Acid-Induced Status Epilepticus Rats. CNS Neurosci. Ther..

[4] Iovino L., Tremblay M.E., Civiero L. (2020). Glutamate-Induced Excitotoxicity in Parkinson’s Disease: The Role of Glial Cells. J. Pharmacol. Sci..

[5] Jeong Y.H., Oh Y.-C., Kim T.I., Bae J.-S., Yeul M.J. (2022). The Neuroprotective Effects of Arecae Pericarpium against Glutamate-Induced HT22 Cell Cytotoxicity. Curr. Issues Mol. Biol..

[6] Gao L., Wang T., Zhuoma D., Yuan R., Huang S., Li B. (2023). Farrerol Attenuates Glutamate-Induced Apoptosis in HT22 Cells via the Nrf2/Heme Oxygenase-1 Pathway. Biosci. Biotechnol. Biochem..

[7] Lee P.J., Pham C.H., Thuy N.T.T., Park H.-J., Lee S.H., Yoo H.M., Cho N. (2021). 1-Methoxylespeflorin G11 Protects HT22 Cells from Glutamate-Induced Cell Death through Inhibition of ROS Production and Apoptosis. J. Microbiol. Biotechnol..

[8] Park D.H., Park J.Y., Kang K.S., Hwang G.S. (2021). Neuroprotective Effect of Gallocatechin Gallate on Glutamate-Induced Oxidative Stress in Hippocampal HT22 Cells. Molecules.

[9] Yao X., Xu X., Hu K., Yang Z., Deng S. (2023). BANF1 Promotes Glutamate-Induced Apoptosis of HT22 Hippocampal Neurons. Mol. Biol. Rep..

[10] Mao X., Wang Z., Zhou H., Liu Z., Zhou Y. (2022). Osthole ameliorates glutamate-induced toxicity in HT22 cells via activating PI3K/Akt signaling pathway. J. Cent. South Univ..

[11] Kritis A.A., Stamoula E.G., Paniskaki K.A., Vavilis T.D. (2015). Researching Glutamate—Induced Cytotoxicity in Different Cell Lines: A Comparative/Collective Analysis/Study. Front. Cell. Neurosci..

[12] Teleanu D.M., Niculescu A.-G., Lungu I.I., Radu C.I., Vladâcenco O., Roza E., Costăchescu B., Grumezescu A.M., Teleanu R.I. (2022). An Overview of Oxidative Stress, Neuroinflammation, and Neurodegenerative Diseases. Int. J. Mol. Sci..

[13] Jaganjac M., Milkovic L., Zarkovic N., Zarkovic K. (2022). Oxidative Stress and Regeneration. Free Radic. Biol. Med..

[14] Sies H. (2015). Oxidative Stress: A Concept in Redox Biology and Medicine. Redox Biol..

[15] Wu Z., Wang H., Fang S., Xu C. (2018). Roles of Endoplasmic Reticulum Stress and Autophagy on H_2_O_2_-induced Oxidative Stress Injury in HepG2 Cells. Mol. Med. Rep..

[16] Forrester S.J., Kikuchi D.S., Hernandes M.S., Xu Q., Griendling K.K. (2018). Reactive Oxygen Species in Metabolic and Inflammatory Signaling. Circ. Res..

[17] Villalpando-Rodriguez G.E., Gibson S.B. (2021). Reactive Oxygen Species (ROS) Regulates Different Types of Cell Death by Acting as a Rheostat. Oxid. Med. Cell. Longev..

[18] Zhao Y., Wang L., Liu M., Du A., Qiu M., Shu H., Li L., Kong X., Sun W. (2023). ROS Inhibition Increases KDM6A-Mediated NOX2 Transcription and Promotes Macrophages Oxidative Stress and M1 Polarization. Cell Stress Chaperones.

[19] Chenna S., Koopman W.J.H., Prehn J.H.M., Connolly N.M.C. (2022). Mechanisms and Mathematical Modeling of ROS Production by the Mitochondrial Electron Transport Chain. Am. J. Physiol.-Cell Physiol..

[20] Zhang Q., Liu J., Duan H., Li R., Peng W., Wu C. (2021). Activation of Nrf2/HO–1 Signaling: An Important Molecular Mechanism of Herbal Medicine in the Treatment of Atherosclerosis via the Protection of Vascular Endothelial Cells from Oxidative Stress. J. Adv. Res..

[21] Li J., Lu K., Sun F., Tan S., Zhang X., Sheng W., Hao W., Liu M., Lv W., Han W. (2021). Panaxydol Attenuates Ferroptosis against LPS-Induced Acute Lung Injury in Mice by Keap1-Nrf2/HO–1 Pathway. J. Transl. Med..

[22] Liu Y., Wang S., Jin G., Gao K., Wang S., Zhang X., Zhou K., Cai Y., Zhou X., Zhao Z. (2023). Network Pharmacology-Based Study on the Mechanism of ShenKang Injection in Diabetic Kidney Disease through Keap1/Nrf2/Ho-1 Signaling Pathway. Phytomedicine.

[23] Tao W., Hu Y., Chen Z., Dai Y., Hu Y., Qi M. (2021). Magnolol Attenuates Depressive-like Behaviors by Polarizing Microglia towards the M2 Phenotype through the Regulation of Nrf2/HO–1/NLRP3 Signaling Pathway. Phytomedicine.

[24] Sun Y.-Y., Zhu H.-J., Zhao R.-Y., Zhou S.-Y., Wang M.-Q., Yang Y., Guo Z.-N. (2023). Remote Ischemic Conditioning Attenuates Oxidative Stress and Inflammation via the Nrf2/HO–1 Pathway in MCAO Mice. Redox Biol..

[25] Wang Y., Gao L., Chen J., Li Q., Huo L., Wang Y., Wang H., Du J. (2021). Pharmacological Modulation of Nrf2/HO–1 Signaling Pathway as a Therapeutic Target of Parkinson’s Disease. Front. Pharmacol..

[26] Xu C., Song Y., Wang Z., Jiang J., Piao Y., Li L., Jin S., Li L., Zhu L., Yan G. (2021). Pterostilbene Suppresses Oxidative Stress and Allergic Airway Inflammation through AMPK/Sirt1 and Nrf2/HO–1 Pathways. Immun. Inflamm. Dis..

[27] Feng Q., Yang Y., Qiao Y., Zheng Y., Yu X., Liu F., Wang H., Zheng B., Pan S., Ren K. (2023). Quercetin Ameliorates Diabetic Kidney Injury by Inhibiting Ferroptosis via Activating Nrf2/HO–1 Signaling Pathway. Am. J. Chin. Med..

[28] Liu G., Wang J., Deng X.-H., Ma P.-S., Li F.-M., Peng X.-D., Niu Y., Sun T., Li Y.-X., Yu J.-Q. (2017). The Anticonvulsant and Neuroprotective Effects of Oxysophocarpine on Pilocarpine-Induced Convulsions in Adult Male Mice. Cell. Mol. Neurobiol..

[29] Zhao P., Chang R.-Y., Liu N., Wang J., Zhou R., Qi X., Liu Y., Ma L., Niu Y., Sun T. (2018). Neuroprotective Effect of Oxysophocarpine by Modulation of MAPK Pathway in Rat Hippocampal Neurons Subject to Oxygen–Glucose Deprivation and Reperfusion. Cell. Mol. Neurobiol..

[30] Zhu Q.-L., Li Y.-X., Zhou R., Ma N.-T., Chang R.-Y., Wang T.-F., Zhang Y., Chen X.-P., Hao Y.-J., Jin S.-J. (2014). Neuroprotective Effects of Oxysophocarpine on Neonatal Rat Primary Cultured Hippocampal Neurons Injured by Oxygen-Glucose Deprivation and Reperfusion. Pharm. Biol..

[31] Yang D., Chen F., Gu Z., Lü L., Ding G., Peng Z., Shang J., Zhang T. (2020). Oxysophocarpine Reduces Oxidative Stress and Inflammation in Tuberculosis-Infected Neutrophils and Mouse Lungs. Int. J. Clin. Exp. Pathol..

[32] Li L., Shi R., Shi W., Zhang R., Wu L. (2020). Oxysophocarpine Protects Airway Epithelial Cells against Inflammation and Apoptosis by Inhibiting miR-155 Expression. Future Med. Chem..

[33] Liu C., Wang R., Jiao X., Zhang J., Zhang C., Wang Z. (2023). Oxysophocarpine suppresses TRAF6 level to ameliorate oxidative stress and inflammatory factors secretion in mice with dextran sulphate sodium (DSS) induced-ulcerative colitis. Microb Pathog..

[34] Shi X., Gao T., Yu C., Fu S., Guo T., Xu W., Li X., Wang Y., Zhang J., Jia X. (2024). Oxysophocarpine attenuates inflammatory osteolysis by modulating the NF-κb pathway and the reactive oxygen species-related Nrf2 signaling pathway. Inflammopharmacology.

[35] Ruera C.N., Perez F., Iribarren M.L., Guzman L., Menendez L., Garbi L., Chirdo F.G. (2023). Coexistence of apoptosis, pyroptosis, and necroptosis pathways in celiac disease. Clin. Exp. Immunol..

[36] Hu Y., Chen D., Hong M., Liu J., Li Y., Hao J., Lu L., Yin Z., Wu Y. (2022). Apoptosis, Pyroptosis, and Ferroptosis Conspiringly Induce Immunosuppressive Hepatocellular Carcinoma Microenvironment and γδ T-Cell Imbalance. Frontiers.

[37] Zhang Y., Wang S., Li H., Xu X. (2021). miR-495 Reduces Neuronal Cell Apoptosis and Relieves Acute Spinal Cord Injury through Inhibiting PRDM5. J. Mol. Histol..

[38] Xu Y.-H., Luo Y., Cao J.-B., Liu Y.-H., Song Y.-X., Zhang X.-Y., Fu Q., Mi W.-D., Li H. (2022). lncRNA BDNF-AS Attenuates Propofol-Induced Apoptosis in HT22 Cells by Modulating the BDNF/TrkB Pathway. Mol. Neurobiol..

[39] Xu D.-H., Du J.-K., Liu S.-Y., Zhang H., Yang L., Zhu X.-Y., Liu Y.-J. (2023). Upregulation of KLK8 Contributes to CUMS-Induced Hippocampal Neuronal Apoptosis by Cleaving NCAM1. Cell Death Dis..

[40] Sun X., Dai L., Zhang H., He X., Hou F., He W., Tang S., Zhao D. (2018). Neuritin Attenuates Neuronal Apoptosis Mediated by Endoplasmic Reticulum Stress In Vitro. Neurochem. Res..

[41] Ketelut-Carneiro N., Fitzgerald K.A. (2022). Apoptosis, Pyroptosis, and Necroptosis—Oh My! The Many Ways a Cell Can Die. J. Mol. Biol..

[42] Sorice M. (2022). Crosstalk of Autophagy and Apoptosis. Cells.

[43] Newton K., Strasser A., Kayagaki N., Dixit V.M. (2024). Cell Death. Cell.

[44] Reactive Oxygen Species-Scavenging Nanosystems in the Treatment of Diabetic Wounds. https://pubmed.ncbi.nlm.nih.gov/37051860/.

[45] Bhatt S., Puli L., Patil C.R. (2021). Role of Reactive Oxygen Species in the Progression of Alzheimer’s Disease. Drug Discov. Today.

[46] Pablo Chapela S., Burgos I., Schiel A., Alonso M., Alberto Stella C. (2021). Serum Reactive Oxygen Species and Apoptosis Markers in Septic Patients. Anaesthesiol. Intensive Ther..

[47] Zuo J., Zhang Z., Li M., Yang Y., Zheng B., Wang P., Huang C., Zhou S. (2022). The crosstalk between reactive oxygen species and noncoding RNAs: From cancer code to drug role. Mol. Cancer.

[48] Sun W., Wang B., Qu X.-L., Zheng B.-Q., Huang W.-D., Sun Z.-W., Wang C.-M., Chen Y. (2019). Metabolism of Reactive Oxygen Species in Osteosarcoma and Potential Treatment Applications. Cells.

[49] Weng Y., Zhang Y., Wang D., Wang R., Xiang Z., Shen S., Wang H., Wu X., Wen Y., Wang Y. (2023). Exercise-Induced Irisin Improves Follicular Dysfunction by Inhibiting IRE1α-TXNIP/ROS-NLRP3 Pathway in PCOS. J. Ovarian Res..

[50] Paradox: Curcumin, a Natural Antioxidant, Suppresses Osteosarcoma Cells via Excessive Reactive Oxygen Species. https://pubmed.ncbi.nlm.nih.gov/37569346/.

[51] Rehfeldt S.C.H., Laufer S., Goettert M.I. (2021). A Highly Selective In Vitro JNK3 Inhibitor, FMU200, Restores Mitochondrial Membrane Potential and Reduces Oxidative Stress and Apoptosis in SH-SY5Y Cells. Int. J. Mol. Sci..

[52] Gorospe C.M., Carvalho G., Herrera Curbelo A., Marchhart L., Mendes I.C., Niedźwiecka K., Wanrooij P.H. (2023). Mitochondrial Membrane Potential Acts as a Retrograde Signal to Regulate Cell Cycle Progression. Life Sci. Alliance.

[53] Zaib S., Hayyat A., Ali N., Gul A., Naveed M., Khan I. (2022). Role of Mitochondrial Membrane Potential and Lactate Dehydrogenase A in Apoptosis. Anticancer. Agents Med. Chem..

[54] Babaei Z., Panjehpour M., Parsian H., Aghaei M. (2022). SAR131675 Receptor Tyrosine Kinase Inhibitor Induces Apoptosis through Bcl- 2/Bax/Cyto c Mitochondrial Pathway in Human Umbilical Vein Endothelial Cells. Anticancer. Agents Med. Chem..

[55] Li Y.-N., Ning N., Song L., Geng Y., Fan J.-T., Ma C.-Y., Jiang H.-Z. (2021). Derivatives of Deoxypodophyllotoxin Induce Apoptosis through Bcl-2/Bax Proteins Expression. Anticancer. Agents Med. Chem..

[56] Huang Y.-K., Chang K.-C., Li C.-Y., Lieu A.-S., Lin C.-L. (2023). AKR1B1 Represses Glioma Cell Proliferation through P38 MAPK-Mediated Bcl-2/BAX/Caspase-3 Apoptotic Signaling Pathways. Curr. Issues Mol. Biol..

[57] Wang R., Song F., Li S., Wu B., Gu Y., Yuan Y. (2019). Salvianolic Acid A Attenuates CCl4-Induced Liver Fibrosis by Regulating the PI3K/AKT/mTOR, Bcl-2/Bax and Caspase-3/Cleaved Caspase-3 Signaling Pathways. Drug Des. Devel. Ther..

[58] Yan H., Huang W., Rao J., Yuan J. (2021). miR-21 Regulates Ischemic Neuronal Injury via the P53/Bcl-2/Bax Signaling Pathway. Aging.

[59] Jiao C., Chen W., Tan X., Liang H., Li J., Yun H., He C., Chen J., Ma X., Xie Y. (2020). Ganoderma Lucidum Spore Oil Induces Apoptosis of Breast Cancer Cells in Vitro and in Vivo by Activating Caspase-3 and Caspase-9. J. Ethnopharmacol..

[60] Araya L.E., Soni I.V., Hardy J.A., Julien O. (2021). Deorphanizing Caspase-3 and Caspase-9 Substrates In and Out of Apoptosis with Deep Substrate Profiling. ACS Chem. Biol..

[61] Unnisa A., Greig N.H., Kamal M.A. (2023). Inhibition of Caspase 3 and Caspase 9 Mediated Apoptosis: A Multimodal Therapeutic Target in Traumatic Brain Injury. Curr. Neuropharmacol..

[62] Batoon L., Koh A.J., Kannan R., McCauley L.K., Roca H. (2023). Caspase-9 Driven Murine Model of Selective Cell Apoptosis and Efferocytosis. Cell Death Dis..

[63] El-Shitany N.A., Eid B.G. (2019). Icariin Modulates Carrageenan-Induced Acute Inflammation through HO–1/Nrf2 and NF-kB Signaling Pathways. Biomed. Pharmacother..

[64] Loboda A., Damulewicz M., Pyza E., Jozkowicz A., Dulak J. (2016). Role of Nrf2/HO–1 System in Development, Oxidative Stress Response and Diseases: An Evolutionarily Conserved Mechanism. Cell. Mol. Life Sci. CMLS.

[65] Ghareghomi S., Moosavi-Movahedi F., Saso L., Habibi-Rezaei M., Khatibi A., Hong J., Moosavi-Movahedi A.A. (2023). Modulation of Nrf2/HO–1 by Natural Compounds in Lung Cancer. Antioxidants.

[66] Duan C., Wang H., Jiao D., Geng Y., Wu Q., Yan H., Li C. (2022). Curcumin Restrains Oxidative Stress of After Intracerebral Hemorrhage in Rat by Activating the Nrf2/HO–1 Pathway. Front. Pharmacol..

